# A CFD-based study on the heat transfer characteristics between the human body and the environment under low-temperature attire

**DOI:** 10.3389/fbioe.2025.1583571

**Published:** 2025-09-09

**Authors:** Shen Yu, Xiaojun Xie, Yan Guo, Jizhe Wang

**Affiliations:** ^1^ State Key Laboratory of Structural Analysis for Industrial Equipment, Dalian University of Technology, Dalian, China; ^2^ Department of Otorhinolaryngology, The Second Affiliated Hospital of Dalian Medical University, Dalian, China

**Keywords:** human numerical model, low temperature, human heat transfer, skin temperature, CFD

## Abstract

In order to study the interaction between the human body, clothing, and environment in a low-temperature environment, a three-dimensional human thermoregulation model was established based on real human body scanning images. The human body was divided into 12 parts by dividing the body into core, equivalent, skin, and clothing layers. Further computational fluid mechanics (CFD) numerical simulation was carried out to realize the measurement and calculation of human physiological parameters, such as human skin temperature, core temperature, and average temperature of the garment surface, in a low-temperature environment. The clothing surface heat transfer, clothing surface average temperature, human skin surface heat transfer, and local skin temperature change rule were explored with the external environmental temperature to establish a basis for the study of the human body’s heat transfer characteristics in a low-temperature environment. To verify the accuracy of the developed numerical model of the human body, the simulated values of the human body model were compared with the experimental measurements of the human local skin temperature and the simulated values in the literature. The results show that the maximum relative error between the local skin temperature of the human numerical model and the experimental measurements of the human body is 3.43%, and the human numerical model has a high degree of accuracy.

## 1 Introduction

Numerical modeling of the human body is a very active research direction that is widely used in aerospace, architectural design, ergonomics, biomedicine, public safety, and other fields. Human body-related research has been greatly promoted in the field of human spaceflight, due to the complexity of the environment in which the mission is carried out, as well as the high requirements for the design of the spacecraft’s environmental module, space suit, and human thermal comfort (Shen et al., 2021). Human numerical modeling also plays an important role in HVAC, in modern biomedicine (obtaining and describing the human body temperature field for medical diagnosis), in human ergonomics (thermal protective equipment design and evaluation), in public safety (guide the emergency rescue, personnel thermal comfort and thermal stress assessment), protective equipment research, and development and testing and so on.

Stolwijk and Hardy divided the human model into three distinctly separate parts: the passive system, the active system, and the clothing system (Stolwijk and Hardy, 1966). The passive system calculates heat exchange within the body and between the body and the environment. The active system actively regulates through the central system, realizing the thermophysiological regulation and control of the human body. The active system and the passive system correspond to the equivalent layer, core layer, and skin layer in the human body model. The clothing system regulates the heat and humidity exchange between the clothing and the environment.

The active system is simplified into three subsystems: sensor, integrator, and effector (Stolwijk and Hardy, 2011). The active system plays an important role in keeping the body temperature stable. The sensors, or thermoreceptors, are located primarily in the skin layer. The integrator system acts as a processing center for thermoreceptor signals, combining and converting these signals into appropriate effector commands. The effector system responds to these commands and translates them into appropriate responses (e.g., vasoconstriction, vasodilation, sweating, and shivering). The human temperature sensing system is controlled by the hypothalamus and is realized by three parts: receptors, central controllers, and effectors, which regulate the body’s thermophysiological system through vasodilatation, vasoconstriction, shivering, and sweating.

Ferreira and Yanagihara (2009) proposed a three-dimensional model to study transient heat transfer in the human body. Havenith and Fiala (2015) built a reliable thermal response model to predict human physiological indicators. Dang et al. (2018) developed an improved three-dimensional model that approximates human body geometry as ellipsoids and ellipsoidal cylinders, which takes into account the core layer of organs, as well as blood flow due to the cardiac cycle. Silva et al. (2018) built a complete 3D thermoregulation model for studying the human body in cold environments but ignored the effect of shivering. Silva et al. (2018) also created a thermoregulation model for hypothermic treatment of neonates. Kang et al. (2019) developed an advanced 3D human thermoregulation model to describe the temperature distribution across the human skin surface and within the body, dividing the human body into 15 parts, and modeling the geometry of human limbs as cones. Cast et al. (2021) developed an anatomically based model of human thermoregulation that predicts the temperature distribution of the entire human body at high temperatures with high spatial resolution. Li et al. (2013) developed a 3D human thermoregulation model based on the actual geometry of a real human body, but they did not take into account the heat generated by shivering and external work and gave less consideration to the effect of clothing. Li et al. (2023) developed a predictive model of the human body in hot environments, which was used to assess the thermophysiological responses and heat stress of personnel in training activities in hot environments.

In recent years, numerical simulation methods have been widely used to study the complex heat flow transport problem of man–clothes–environment, especially those based on computational fluid mechanics (CFD). Most of the studies are on ambient temperatures between 10 °C and 40 °C, with relatively few studies on ambient temperatures between 0 °C and 10 °C. In this article, a simple 3D human thermoregulation model is developed based on human body images and real human body boundaries. The heat and mass transfer characteristics of the human body at low ambient temperatures of 0 °C–14 °C were investigated. This model has the potential application to study human thermal comfort and the performance of clothing systems in many research fields such as built environments, functional clothing design and engineering, ergonomics, and environmental medicine.

## 2 Methodology

### 2.1 Model geometry

**FIGURE 1:**
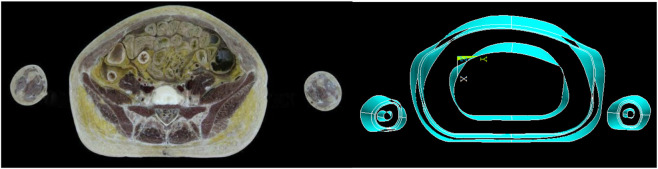
Example of a single-layer scan image of the human body (left), from the innermost core layer to the outermost layer, equivalent layer, skin layer, and clothing layer (right).

**FIGURE 2:**
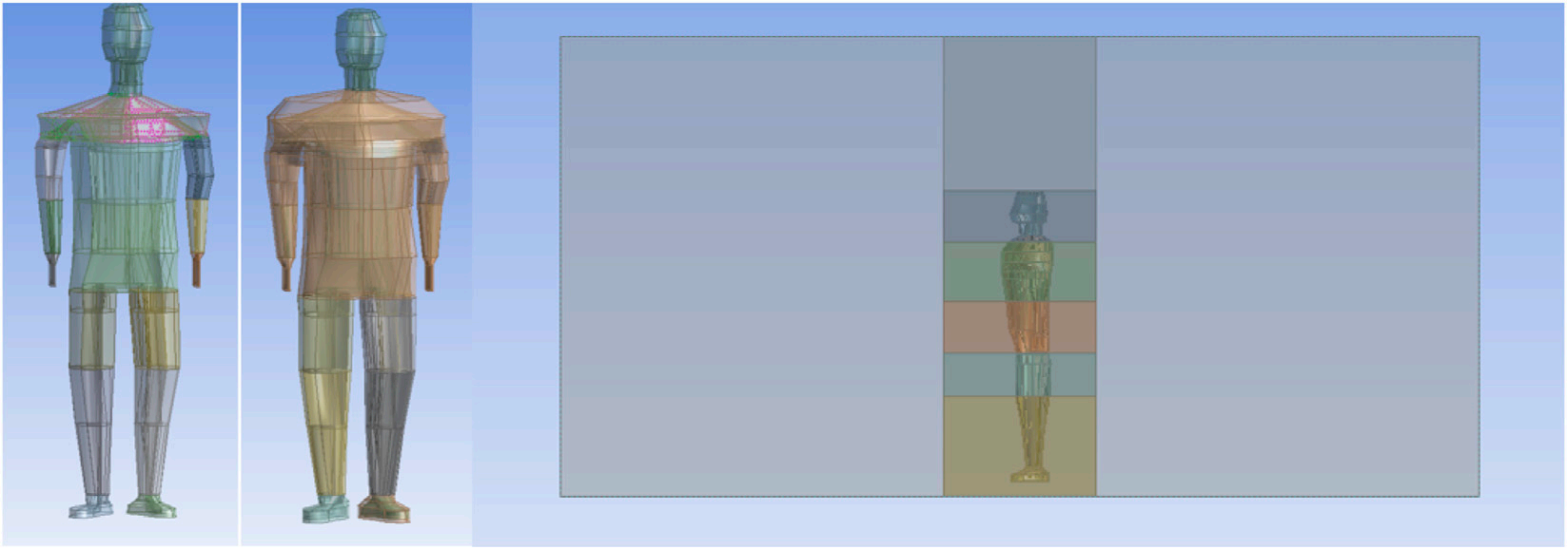
Segmentation of the unclothed human body, segmentation of the clothed human body, and computational domain segmentation.

The numerical model of humans used in this study is based on the real human body model. Based on the real human body image, the human body is scanned at a certain interval to get a color image. The number of participants in the experiment is n = 1. The real human body is based on a Chinese healthy adult man, aged 25 years old, with a height of 178 cm, a weight of 80 kg, and a human skin surface area of 1.92 m^2^. The color image was grayscaled using MATLAB to obtain the boundary 3D coordinates (x, y, z) of the image. Coordinate points were entered into the Ansys Parametric Design Language (APDL) command stream to obtain the boundaries of the single-layer human image. The model was then modeled in APDL using tools such as masking and tangents. As shown in Figure 1, from the inside to the outside, they are the core layer, the equivalent layer, the skin layer, and the clothing layer. The human body model is built based on irregular surfaces. It is quite difficult to model the ends of the limbs, the hands, and the feet into four layers, and model simplification is performed for these parts. Finger and toe parts are modeled and simplified. The modeling of the axillary and crotch parts must also be simplified. The final 3D model of the human body is a simplified model based on the contours of the real human body. The above obtained surface model was imported into ANSYS for human model filling division and computational domain establishment. As shown in Figure 2, the human body model is segmented into 12 anatomical regions based on geometric topology: head, shoulders, chest, abdomen, upper arms, forearms, hands, left thigh, right thigh, left calf, right calf, and bilateral feet.

### 2.2 Governing equations

The heat balance equations for the layers of each part of the body and the central blood compartment are given in the following equation (see Equations 1–4) (Tanabe et al., 2002).

Core layer:

$$C_{p}\left(i,1\right)\frac{dT\left(i,1\right)}{dt}=Q\left(i,1\right)-Q_{b}\left(i,1\right)-Q_{c}\left(i,1\right)-RES\left(i,1\right)$$ (1)

Equivalent layer :

$$C_{p}\left(i,2\right)\frac{dT\left(i,2\right)}{dt}=Q\left(i,2\right)-Q_{b}\left(i,2\right)+Q_{c}\left(i,1\right)-Q_{c}\left(i,2\right)$$ (2)

Skin layer:

$$C_{p}\left(i,3\right)\frac{dT\left(i,3\right)}{dt}=Q\left(i,3\right)-Q_{b}\left(i,3\right)+Q_{c}\left(i,2\right)-Q_{c}\left(i,3\right)-E\left(i,3\right)$$ (3)

In the above equation, C $\left(J\cdot kg^{-1}\cdot K^{-1}\right)$ is the heat capacity of each node of the human body. T °C*_p_* is the temperature of each node of the human body. t(h) is the time of exposure to the environment. Q (i,j)(W) is the heat production of each part. Q_b_ (i,j) (W)is the heat exchange of blood (W). Q_c_ (i,j) (W)is the conduction heat exchange between different layers in the same part of the human body. RES (i,j) (W) is the respiratory heat transfer. E (i,j) (W)is the evaporative heat transfer of skin (W). Q_r_ (W)is convective and radiative heat transfer.

The temperature of blood in the capillaries and veins of the central blood layer is equal to the temperature of the neighboring tissues. The heat balance of heat exchange between the central blood layer and the various nodes of the body’s layers is described by the following equation. In this article, the heat exchange of the central blood layer is calculated and added to the equivalent layer.

$$C_{p,b}\frac{dT}{dt}=\sum_{i=1}^{k}\sum_{j=1}^{n}Q_{b}\left(i,j\right)$$ (4)

The body’s heat production consists of three components: basal metabolic heat production, external work, and shivering heat production. External work and shivering heat production only occur in the equivalent layer; the other layers are 0 (see Equation 5).

$$Q\left(i,j\right)=Q_{0}\left(i,j\right)+W\left(i,j\right)+Ch\left(i,j\right)$$ (5)

Q_0_ (i,j)(W) is the sum of basal metabolic heat production, which is the amount of heat produced by each part of the body. W (i, j) is the external work. Ch(i, j) is the heat generated by shivering (Tanabe et al., 2002).

The amount of change in metabolism at low temperatures is calculated in the following equation (see Equation 6):

$$Q^{\text{∗}}\left(i,j\right)=Q\left(i,j\right)-\Delta Q\left(i,j\right)$$ (6)

In the above equation, Q^∗^(i,j) is the metabolic rate of the human body in a low-temperature environment. ΔQ (i,j) is the amount of change in basal metabolic rate at low temperatures and represents the effect of temperature on physiological responses. It is a function of the temperature difference between the node temperature and its set point temperature. ΔQ (i,j) is a function of the difference between the temperature of each layer of each block and the temperature of the turning point. As shown in Equation 7 (Werner and Buse, 1988), when the body is in a thermal neutral state, where energy is supplied by the basal metabolic rate, the temperature of each part of the body is called the tempering point temperature.

$$\Delta Q\left(i,j\right)=Q_{0}\left(i,j\right)\left[2^{0.1\left(T-T_{0}\right)}-1\right]$$ (7)

At low temperatures, external work and shivering simultaneously generate heat, and the heat generated occurs only in the equivalent layer (j = 2). The heat generated in the other layers is $W\left(i,j\right)=Ch\left(i,j\right)=0$ . According to Equation 8,

$$W\left(i,2\right)=58.2\left(met-Q_{0}\right)AMetf\left(i\right)$$ (8)

A is the surface area of each part of the human body, and when W (i,2) is a negative value, it can be considered zero. met is the body’s metabolic heat production and can be obtained from the ISO standard. Metf (i) is the coefficient of distribution of muscle heat production between different layers in different zones of the body. Q_0_ is obtained by summing the basal metabolic rates of the nodes.

Thermal convection is the macro-motion of the fluid caused by the relative displacement between the various parts of the fluid. Cold and hot fluids mix with each other as a result of the heat transfer process. The central blood layer exchanges heat with the blood in a convective manner through all parts of the body and plays a very important role in the process of thermoregulation of the body. See Equation 9:

$$B=a\rho B_{f}\left(T-T_{bf}\right)$$ (9)

a is the countercurrent heat transfer ratio, a = 1.000 (Tanabe et al., 2002); ρ is the specific heat of blood by volume. ρ=1.067Wh/l·Co . B_f_ (m/s)is the blood flow rate.

In a low-temperature environment, the central nervous system controls capillary contraction. Blood flow is greatly reduced, thereby reducing the body’s heat dissipation to the environment. Blood flow is calculated as follows (see Equation 10):

$$B_{f}^{*}=B_{f}-\Delta B_{f}$$ (10)

B*_f_ is the blood flow in the human body at low temperatures. ΔB_f_ is the amount of change in blood flow triggered by hypothermia. The expression is as follows (see Equation 11) (Werner and Buse, 1988):

$$\Delta B_{f}=B_{b}\cdot \left[{2^{\left(T-37\right)}}^{/10}-1\right]$$ (11)

It is generally accepted that respiratory heat exchange occurs primarily in the lungs and only at the core of the chest. It is proportional to the volume of respiration and therefore to total metabolic energy production. Respiratory heat loss is shown in Equation 12.

$$R_{resp}=\left\{0.0014Q_{tot}\left(34-T_{a}\right)+0.017Q_{tot}\left(5.867-P_{a}\right)\right\}$$ (12)

The body’s thermophysiological regulation is achieved through the regulation of the thermal response by the hypothalamus in the central nervous system. Temperature perception is characterized by the body temperature difference signal error, as shown in Equation 13 (Tanabe et al., 2002).

$$Err=\left(T-T_{set}\right)+R_{ate}F=\left(T-T_{set}\right)$$ (13)

The hot signal W_arm_ and the cold signal C_old_ correspond to hot and cold receivers. When the condition Err>0 holds, it is expressed by Equation 14, and when the condition Err<0 holds, it is expressed by Equation 15.

Warm=Err,Cld=0 (14)

Cld=−Err,Warm=0 (15)

Based on the calculation of the hot and cold signals of the skin temperature, a comprehensive signal of the human body temperature perception can be obtained. Using the signals from the skin temperature receptors as control variables, the integrated heat signal and the integrated cold signal are represented by Equations 16, 17, respectively, in the formula. The weight distribution coefficient SKINR (i) is shown in the literature (Tanabe et al., 2002).

$$Warms=\sum_{i=1}^{13}\left(SKINR\left(i\right)\times Warm\left(i,3\right)\right)$$ (16)

$$Clds=\sum_{i=1}^{13}\left(SKINR\left(i\right)\times Cld\left(i,3\right)\right)$$ (17)

The human thermoregulatory system consists of four components (vasoconstriction, vasodilation, heat production from chills, and evaporation of sweat). In cold environments, vasoconstriction reduces heat loss, and shivering generates heat to maintain body temperature. In a hot environment, vasodilation and sweating work together to dissipate heat and maintain body temperature.

The human skin blood flow BF (i, 3) can be calculated from Equation 18:

$$BF\left(i,3\right)=\frac{B_{b}+\left(SKINV\left(i\right)\times D_{L}\right)}{1+\left(SKINC\left(i\right)\times S_{T}\right)}\times km\left(i,3\right)$$ (18)

Where km (i,3) is a regional multiplier reflecting the effect of local skin temperature on vasodilation and vasoconstriction. Equations 19–21 present the calculations. In this article, RT (i,3) = 10 °C.

$$km\left(i,3\right)=2.0^{Err\left(i,3\right)/RT\left(i,3\right)}$$ (19)

$$D_{L}=C_{dl}Err\left(1,1\right)+S_{dl}\left(Wrms-Clds\right)+P_{dl}Wrm\left(1,1\right)Wrms$$ (20)

$$S_{T}=-C_{st}Err\left(1,1\right)-S_{st}\left(Wrms-Clds\right)+P_{st}Cld\left(1,1\right)Clds$$ (21)

In the above equation, D_L_ is the vasodilation signal, and S_T_ is the vasoconstriction signal.

### 2.3 Boundary conditions

The left boundary of the computational domain is designated as the velocity inlet, and the right boundary is designated as the pressure outlet. The surrounding sidewalls are set as adiabatic stationary boundary conditions, and the entire computational domain: L = 6 m, W = 3 m, and H = 3 m. The feet of the 3D mannequin are close to the ground. The human numerical model is in the middle of the computational domain. The core layer of the human body model is hollow, and the inner surface of the core layer is set as a constant 37 °C isothermal wall surface, thereby simulating the core body temperature of a human being. The outer surface of the skin and the inner surface of the garment are considered to be in ideal contact, ignoring the air gap between them (Shen et al., 2018). Therefore, the heat from the skin is transferred to the clothing layer mainly by heat conduction. The outer surface of the garment is in contact with the fluid domain. The air in the fluid domain is considered incompressible air.

The 3D human model is divided into four layers: the core layer, the equivalent layer, the skin layer, and the clothing layer. The thermodynamic parameters for each layer are taken from Werner and Buse (1988). Table 1 shows the equivalent physical property parameters of each part of the human body.

**TABLE 1:** Physical parameters of equivalent layers in various parts of the human body (Tanabe et al., 2002).

Body part	Head	Shoulder	Upper arm	Lower arm	Chest	Abdomen	R-thigh	L-thigh	R-calf	L-calf
Thermal conductivity W/(m×k)	0.1	0.8	0.2	0.1	0.1	0.2	0.05	0.05	0.12	0.12
Specific heat capacity J/(Kg×K)	2318.4	1678.6	1764	1861.2	1547	2219.7	1000	1000	2354.4	2354.4

### 2.4 Grid division

**FIGURE 3:**
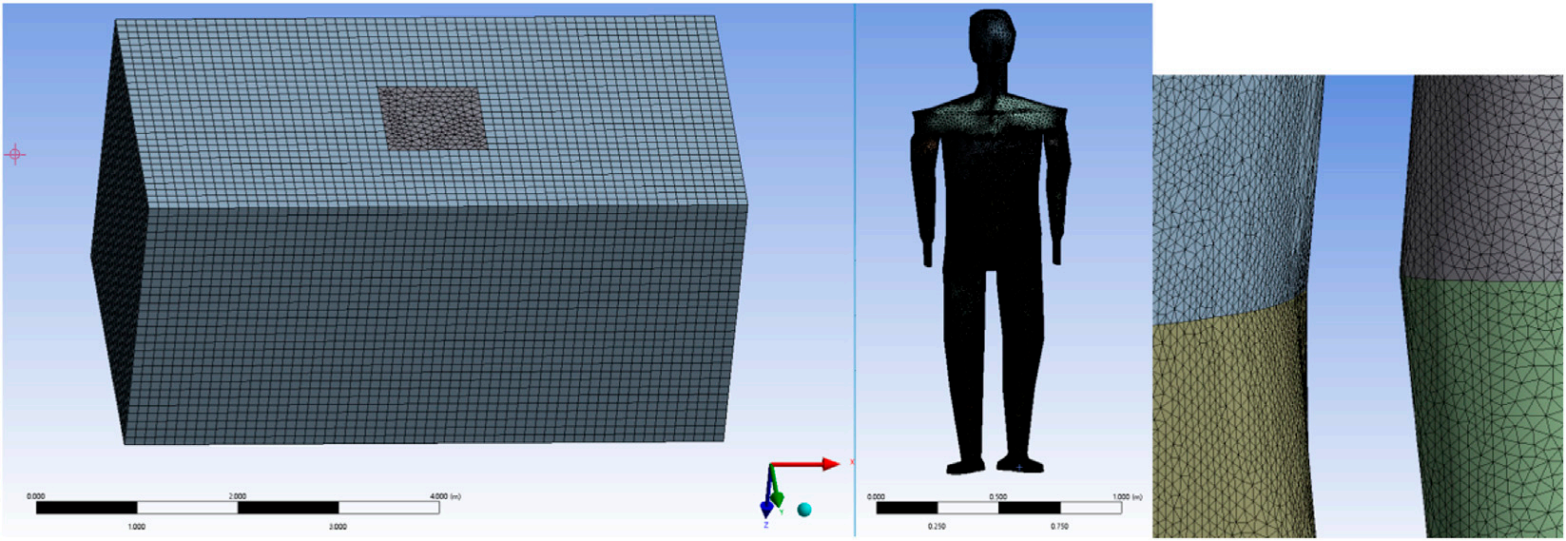
Computational domain and human model mesh dissection.

In Figure 3, the outermost mesh is dissected using a structured mesh. The human body model is built as an irregular surface. Because it is difficult to dissect the human body model with a structured mesh, the mesh of the human model is dissected using an unstructured mesh. Immediately adjacent to the garment, the velocity and temperature fields of the computational domain are more variable, and the mesh is encrypted near the outer surface of the garment. The mesh contains about 6,550,000 cells and 1,160,000 nodes in total. The heat flow transfer involves the solution of the continuity, momentum, and energy equations, which are solved in this article using the finite volume method (FVM) of the commercial CFD software Ansys-Fluent.

### 2.5 Measurement of physical parameters of clothing and skin temperature

A human local skin temperature thermometry experiment was set up to verify the accuracy of the human numerical model. The accuracy of human numerical models was also verified by setting up experiments to measure physical parameters of garments.

The temperature measurements employed Wodsun (WDS) button-type data loggers. Technical specifications of the devices are provided in Table 2. Following proper donning of all 12 sensors, regional skin temperatures were sampled at 1-min intervals throughout the experimental protocol.

**TABLE 2:** Experimental equipment selection parameters.

Parameter	Number of sensors	Sample interval	Sampling time	Temperature accuracy	Temperature measurement range
	12	1 min	60 min	±0.05C	−40 °C−+85 ° C

To verify the accuracy of the numerical human model, local skin temperature measurement experiments and clothing physical property parameter measurement experiments were established to verify the human numerical model. Prior to the experiments, the cold-weather suits and undergarments were pretreated at an ambient temperature of 20 °C and a relative humidity of 65% for 24 h to bring their temperature and humidity to a dynamic equilibrium state (Tu et al., 2020; Tu et al., 2021).

The inner and outer fabric of the garment is 100% polyester fiber. The thermal conductivity, specific heat capacity, and permeability of a garment that has not been worn are very different from those of a garment that has been washed many times. Therefore, this experiment uses garments that have been worn and washed many times to simulate attire in a real environment.

The experiment for measuring physical parameters of garments is based on the TC3200 thermal conductivity meter. Measurements were made using the transient hot wire method. The transient method means that the sample under test is measured as the temperature changes. The transient method has a shorter measurement time (1 s to a few minutes), is more accurate, and only requires good contact for the physical model. The temperature rise of the hot wire in the transient hot wire method is a primary function of the natural logarithm of the heating time. The thermal conductivity can be calculated by using the $\Delta T_{d}∼\ln\tau$ -curve with the functional equation. The fundamental equation is (Low cost and new design, 2024) (see Equation 22).

$$\lambda =\frac{q}{4\pi }/\frac{\Delta T}{\ln\tau }$$ (22)

q $\left(W\cdot m^{-1}\right)$ is the heating power per unit length of the hot wire source. $\Delta T\left(K\right)$ is the change in temperature of the hot wire. τ(s) is the heating time of the hot wire.

The experimental clothing is divided into two categories: summer clothing and cold-weather clothing. Because the measurement of physical parameters of garments is a destructive experiment, two sets of the same garments were prepared. One set is used for clothing physical property parameter measurement experiments, and another set is used for human local skin temperature measurement experiments. There are three choices of measurement points for cold-weather clothing. 1. The left and right chest garments are symmetrical and serve as one test point. 2. Upper arm, lower arm, and back are the same thickness and serve as one test point. 3. Left and right abdominal garments are the same thickness and serve as one test point. The thickness of the sample at the three test points was measured using a vernier caliper to determine the thermal conductivity at the three test points. Measurements were repeated five times for each sample, and the data were averaged. The thermal conductivity of clothing was calculated with reference to the average skin temperature (Qian and Fan, 2006). The static weighted thermal conductivity is calculated using Equations 23, 24. The final garment’s physical property parameter measurements are shown in Table 3. The thermal conductivity of the upper half of the cold-weather clothing is not much different from the 0.0657 w/(m·k) measured by Shen et al. (2021).

$$\lambda_{tot-st-up}=\left(\lambda_{back}+\lambda_{Abdomen}\right)\times 0.101+\lambda_{chest}\times 0.195+\lambda_{abdomen}\times 0.128+\lambda_{buttock}\times 0.136+\lambda_{upper-arm}\times 0.21+\lambda_{Lower-arm}\times 0.128$$ (23)

$$\lambda_{tot-st-down}=\lambda_{Thigh}\times 0.524+\lambda_{\mathrm{calf}}\times 0.476$$ (24)

**TABLE 3:** Physical parameters of winter clothing and summer clothing.

Physical parameters of clothing	Summer clothing	Winter clothing
Thermal conductivity of the upper body w/(m·K)	0.034	0.062
Thermal conductivity of the lower body w/(m·K)	0.031	0.037
Specific heat capacity of the upper body kj/(kg·k)	0.648	0.161
Specific heat capacity of the lower body kj/(kg·k)	0.375	0.282
Upper body thickness (mm)	0.4	5.5
Lower body thickness (mm)	0.9	5.1

The formula for calculating thermal resistance are as follows (see Equations 25–27):

$$R_{t}=\frac{A\left(T_{s}-T_{a}\right)}{q_{avg}}$$ (25)

$$R_{a}=\frac{A\left(T_{c}-T_{a}\right)}{q_{avg}}$$ (26)

$$R_{i}=R_{t}-\frac{R_{a}}{f_{cl}}$$ (27)

In the above equation, R_t_ is the total thermal resistance of the surface air layer, R_i_ is the intrinsic thermal resistance, and R_a_ is the thermal resistance. T_s_, T_c_, and T_a_ ( °C) are the skin surface temperature, the outer surface temperature of clothing, and the ambient temperature. $q_{avg}$ is the steady-state mean heat flow density from the body to the environment. A represents the surface area of the hot mannequin (m^2^). $f_{cl}$ is the dimensionless clothing area factor. According to previous studies, the area factor is 1.35.

## 3 Results and discussion

### 3.1 Model verification

Local skin temperature measurement experiments and numerical simulations were carried out, taking two external experimental environments as examples (natural environment, indoor environment). Then, the accuracy of the 3D human body numerical model was verified. The heat production of the active system and the heat production of the passive system were calculated using the equations above, and the obtained heat production was entered into the equivalent layer. The skin temperature of each part of the human body obtained from the numerical model was compared with the human body’s local skin temperature measurements. At the same time, the convective heat exchange in the steady state environment was calculated, and the corresponding heat exchange coefficients were obtained.

$$Q_{c}=h_{c}\left(T_{sk}-T_{a}\right)A_{sk}$$ (28)

In the above formula (see Equation 28), Q_c_ (W) is the convective heat transfer capacity. h_c_ $w/\left(m^{2}\cdot K\right)$ is the convective heat transfer coefficient. T_sk_ (°C) is the numerical dummy skin temperature. T_a_ (°C) is the ambient air temperature. A_sk_ (m^2^) is the area of each part of the numerical human model.

**FIGURE 4:**
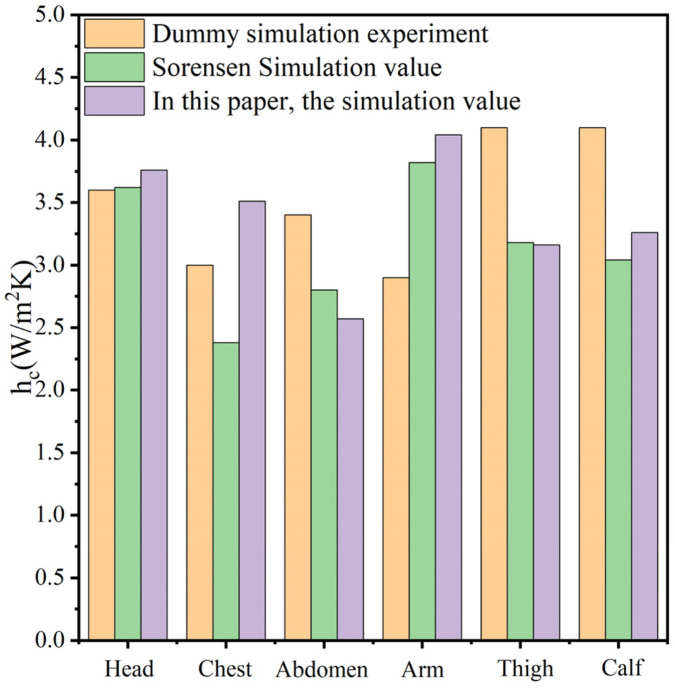
Comparison of convective heat transfer coefficient with de Dear and Sorensen.

First, the experimental environment was verified. The indoor temperature was 18 °C, the relative humidity was 50%, and the subject human body was wearing short sleeves and shorts. CFD calculates the heat transfer coefficients of each part of the human body and compares the obtained heat transfer coefficients with the results of the Sørensen and Voigt (2003) simulation and the values obtained from the experiments of De Dear et al. (1997) dummy to verify the accuracy of the human body model. The CFD boundary condition settings for this case are approximately the same as the de Dear dummy experiment and the Sorensen calculation case. The outside temperature in Sorensen is 19.75 °C. The results of the comparison are shown in Figure 4. The model in this article has less deviation in the values of heat transfer coefficients for head, thigh, calf, and abdomen compared to Sorensen. However, these sites were more deviated than the de Dear dummy measurements. The main reason for such a deviation is that the experiments with Dear's dummy were carried out in a wind tunnel where the wind was blowing horizontally and uniformly. In this working condition CFD simulation, the wind speed in the computational domain is controlled at 0 m/s. Another aspect is that the dummy model’s attire is less than short sleeves and shorts, and in this article, there is no clothing covering the lower arms and legs of the human body. The convective heat transfer coefficients obtained for each part of the body were weighted with the area of each part and the area of the body. The final calculated overall convective heat transfer coefficient for the human body was 3.27 $w/\left(m^{2}\cdot K\right)$ . This is closer than the 3.13 $w/\left(m^{2}\cdot K\right)$ calculated by Sørensen and Voigt (2003). This is closer to the De Dear et al. (1997) average natural convection heat transfer coefficient of (3.3 $w/\left(m^{2}\cdot K\right))$ also.

The local average skin temperature values were then measured for each of the 12 parts of the human body under the external natural environment of 0 °C, 3 °C, 5 °C, 8 °C, 12 °C, and 14 °C, respectively. The human numerical model was validated for local boundary temperatures because the local average skin temperature of the human body is related to human metabolism, and the metabolism is related to the external environment, the physical parameters of the worn clothing, the characteristics of the human body (fat content, heart rate), and the time of day (Stolwijk and Hardy, 1966). We fixed the experiment of human skin temperature measurement at the same place and in the same time period (18:00–19:00) and used the measured values after the sensor stabilization. Prior to 18:00 h, subjects were instrumented with sensors and donned properly fitted protective garments. All participants abstained from food intake and vigorous exercise for 30 min preceding the experiment. At precisely 18:00, subjects entered either natural outdoor environments or controlled climatic chambers. Throughout testing, participants maintained stationary positions with minimal movement. Measurements were recorded exclusively during post-stabilization periods of sensor readings. All trials occurred during nighttime conditions when ambient radiative heat flux was negligible; therefore, environmental radiation exchange was excluded from thermal analyses.

Because the human body is affected by the temperature and humidity of the surrounding external environment, there are small fluctuations in the value of the local skin temperature of the human body. We used the average of (30) stable values over the measurement time period to exclude the effects of small environmental factors and experimental data errors in the symmetrical parts of the mannequin (left and right abdomen, left and right chest, left and right thighs, and left and right calves) due to minor differences in the external environment, differences in human metabolism, and differences in the distribution of tissues within the body. This ultimately leads to variability in body temperature values at symmetrical sites. The average temperature difference of the measured values is within 2 °C.

**FIGURE 5:**
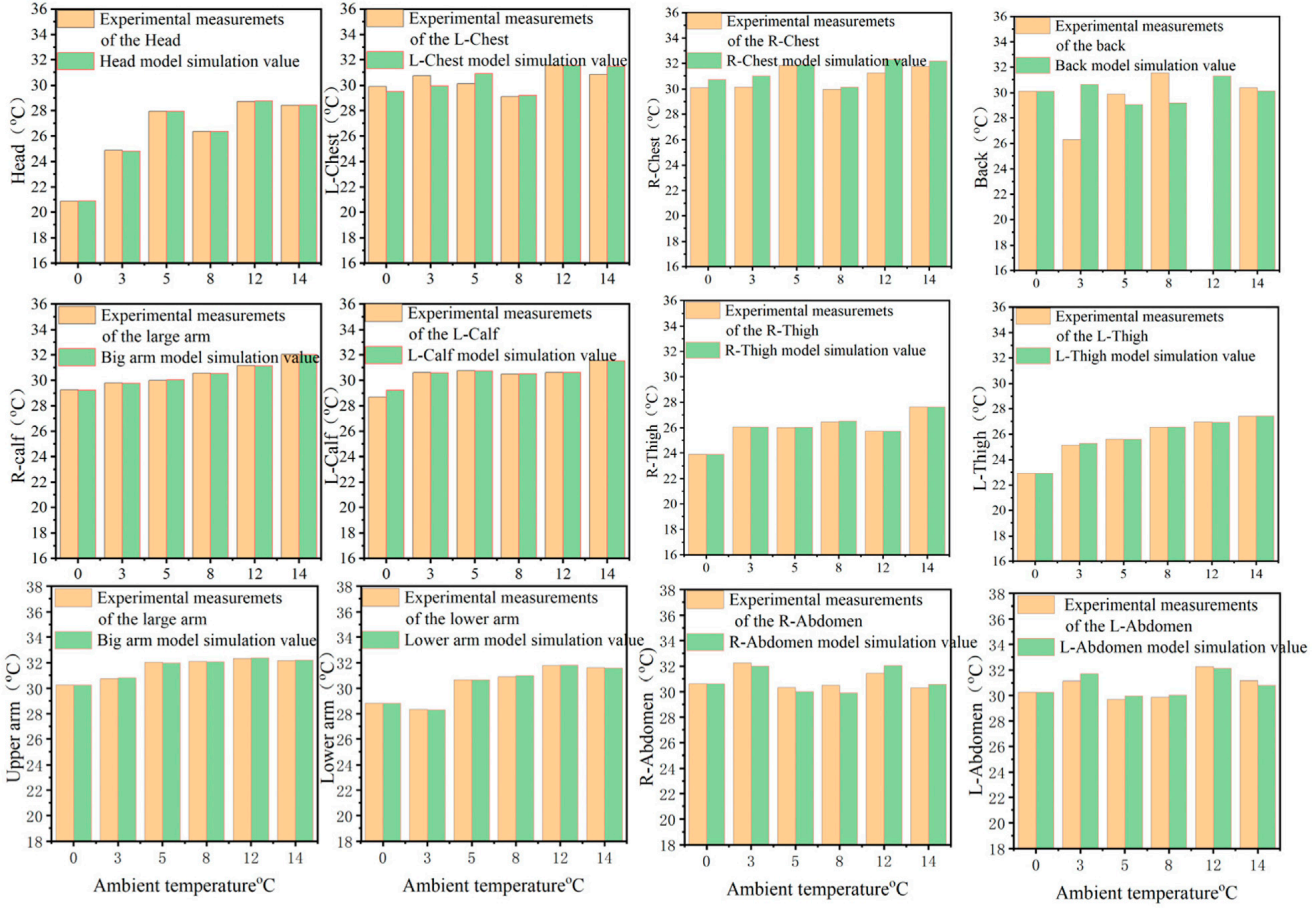
Comparison of simulated and experimentally measured values of human local skin temperatures in different external environments for the same garment.

**FIGURE 6:**
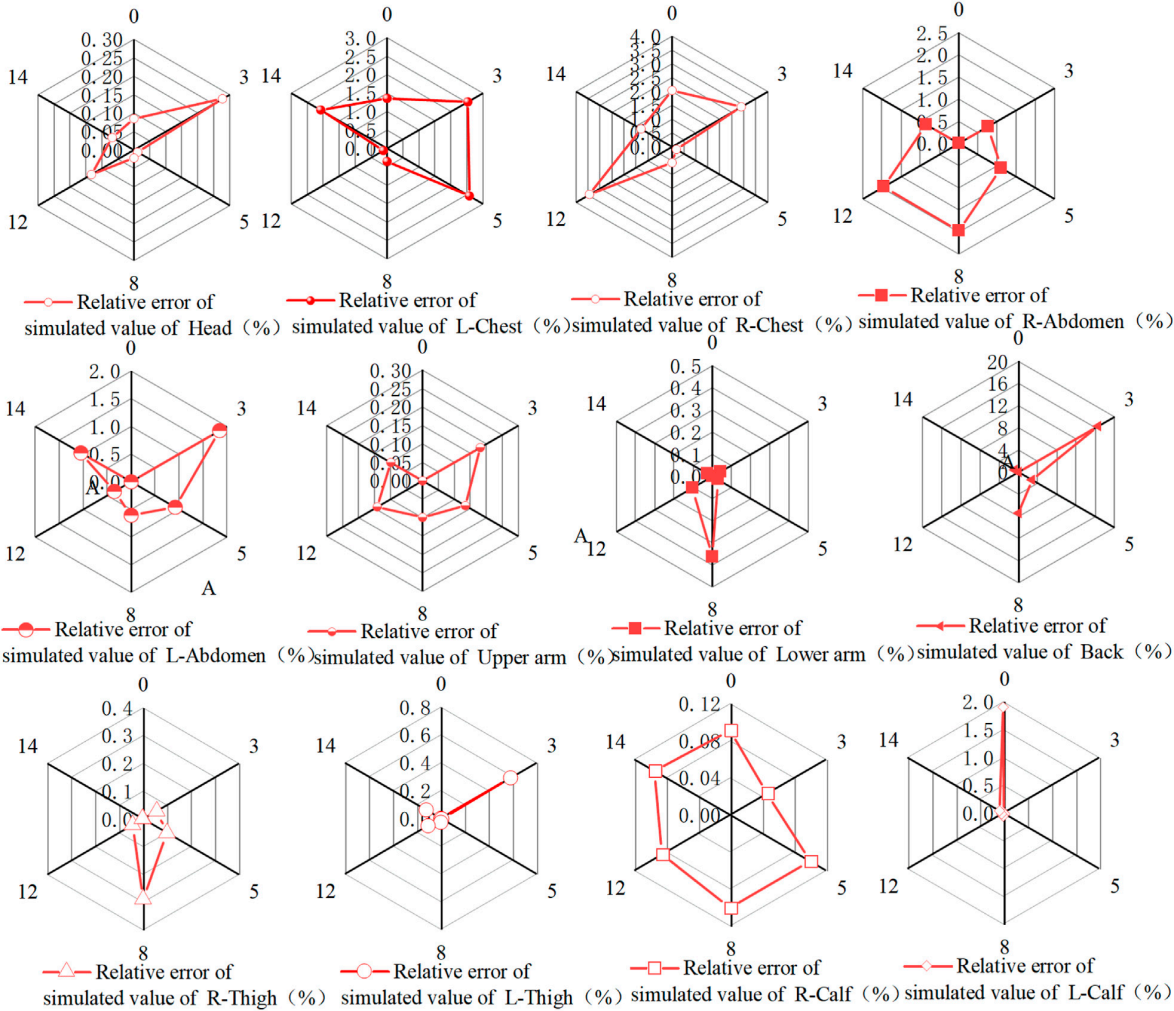
Relative error between simulated and experimentally measured skin temperatures in various parts of the human body.

The numerical simulation results are shown in Figure 5. The same material properties, core layer boundary conditions, and solution method are entered under the same dress code and in different external environments. The heat source sizes of different equivalent layers for skin local boundary temperature coupling are entered to obtain the relative error between the simulated values and the experimental measurements of human local skin temperature. The relative errors under external ambient conditions of 0 °C, 3 °C, 5 °C, 8 °C, 12 °C, and 14 °C are shown in Table 4. The relative error of the chest is greater because of the asymmetrical distribution of tissues within the chest. The relative error of the local skin temperature of the human body model is larger when the left and right abdomen and back are divided into parts. At the same time, there is a slight difference between the thickness of the clothing layer and the actual thickness of the worn garment, which is also a source of relative error. The value of the relative error of the average human body temperature is maximum at 3 °C of the external ambient temperature, and the maximum value of the relative error is 5.35%. As shown in Figure 6, the value of the relative error of the numerical simulation is smaller and the accuracy of the numerical model of the human body is higher closer to the center point. The simulated values of local skin temperature in most parts of the body are coupled with experimental measurements with high accuracy, and overall, the established 3D human body numerical model has high accuracy and reliability in different external environments.

**TABLE 4:** Relative error between simulated and experimentally measured values of human local skin temperature.

Environmental temperature (°C)	Head	Upper arm	Lower arm	L-Chest	R-Chest	L-Abdomen	R-Abdomen	L-thigh	R-thigh	L-calf	R-calf	Back
0 °C	0.085%	0.00%	0.00%	2.04%	1.35%	0.00%	0.00%	0.00%	0.00%	0.09%	1.91%	0.00%
3 °C	0.28%	0.18%	0.043%	2.88%	2.52%	0.76%	1.85%	0.057%	0.58%	0.05%	0.01%	16.58%
5 °C	0.012%	0.133%	0.028%	0.18%	2.57%	1.10%	0.92%	0.10%	0.01%	0.10%	0.032%	2.79%
8 °C	0.022%	0.10%	0.36%	0.57%	0.35%	1.97%	0.61%	0.29%	0.03%	0.10%	0.058%	7.37%
12 °C	0.13%	0.14%	0.10%	3.44%	0.11%	1.96%	0.35%	0.04%	0.11%	0.08%	0.032%	6.54%
14 °C	0.067%	0.01%	0.02%	1.30%	2.08%	0.85%	1.04%	0.00%	0.12%	0.09%	0.08%	0.83%

### 3.2 Thermal resistance and clothing surface heat transfer

**FIGURE 7:**
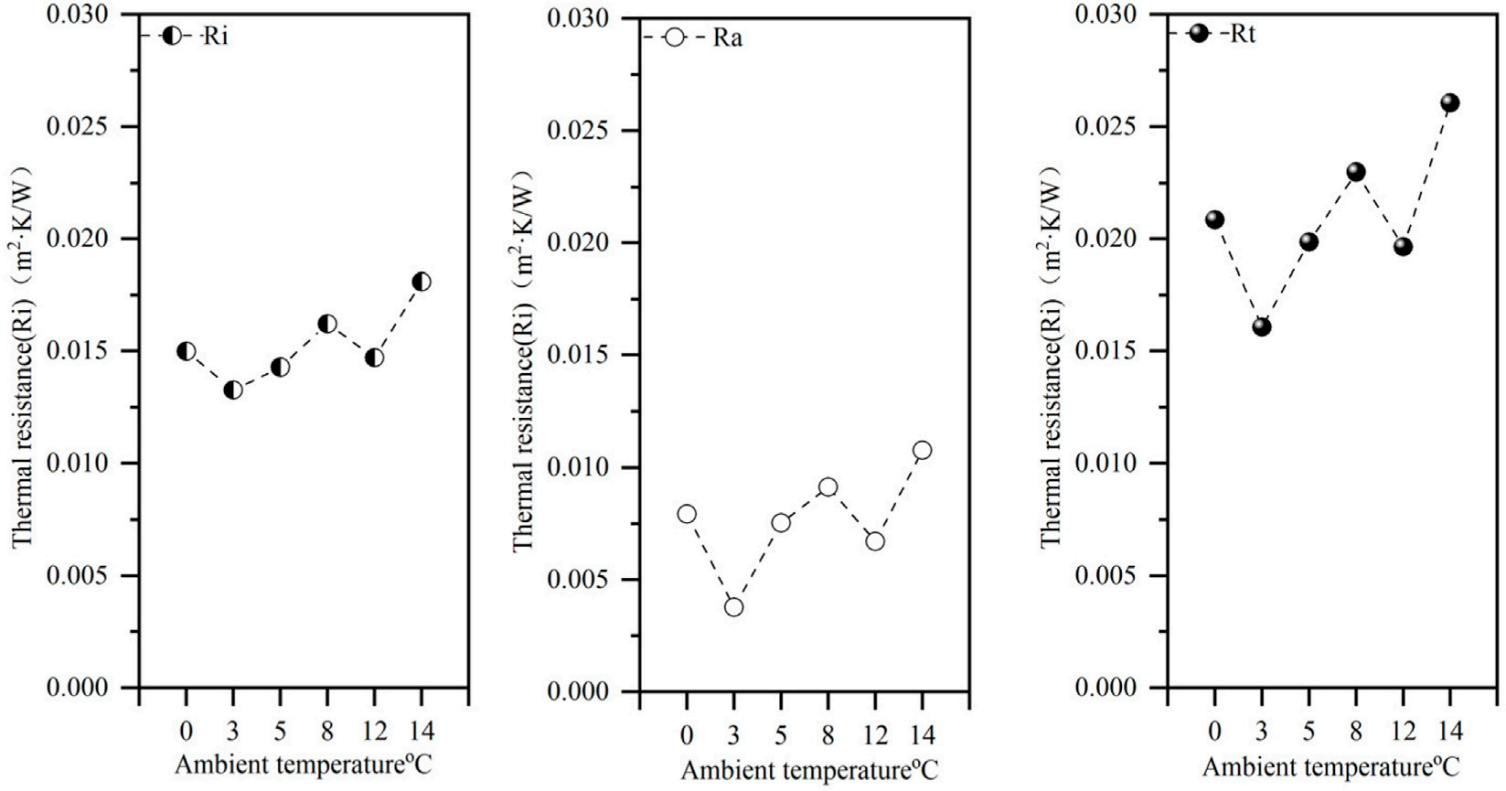
Human body CFD simulation of thermal resistance (thermal resistance Rt, inherent thermal resistance of the garment Ri, and thermal resistance of the enclosed air layer Ra).

The amount of heat exchange in a garment is not only related to its thickness, but also to the outside ambient temperature (Shen et al., 2021). In Figure 7, the external ambient temperatures are 0 °C, 3 °C, 5 °C, 8 °C, 12 °C, and 14 °C, and the total thermal resistance Rt, the intrinsic thermal resistance of the garment Ri, and the thermal resistance of the surface closed air layer Ra are obtained by numerical simulation methods. According to previous research, the additional thermal resistance added to the very thin air layer on the outer surface of the garment plays an important role in reducing the heat flux into the surrounding environment (Havenith et al., 1990). The numerical results show that under the same garment, with the change in external ambient temperature, the value of thermal resistance Ra of the closed air layer fluctuates in the range of 0.00378–0.01075 m^2^·K/W, the intrinsic thermal resistance Ri of the garment fluctuates in the range of 0.01327–0.01810 m^2^·K/W, and the thermal resistance Rt fluctuates in the range of 0.01607–0.02606 m^2^·K/W, under common human body and external environment conditions. The percentage of Ra in the overall thermal resistance ranges from 23% to 41%, which indicates that Ri contributes more to body insulation. As shown in Figure 7, for clothing inherent thermal resistance Ri, thermal resistance Rt, and closed air layer thermal resistance Ra have a small resistance fluctuation range with increasing external ambient temperature. The resistance value remains relatively stable.

**FIGURE 8:**
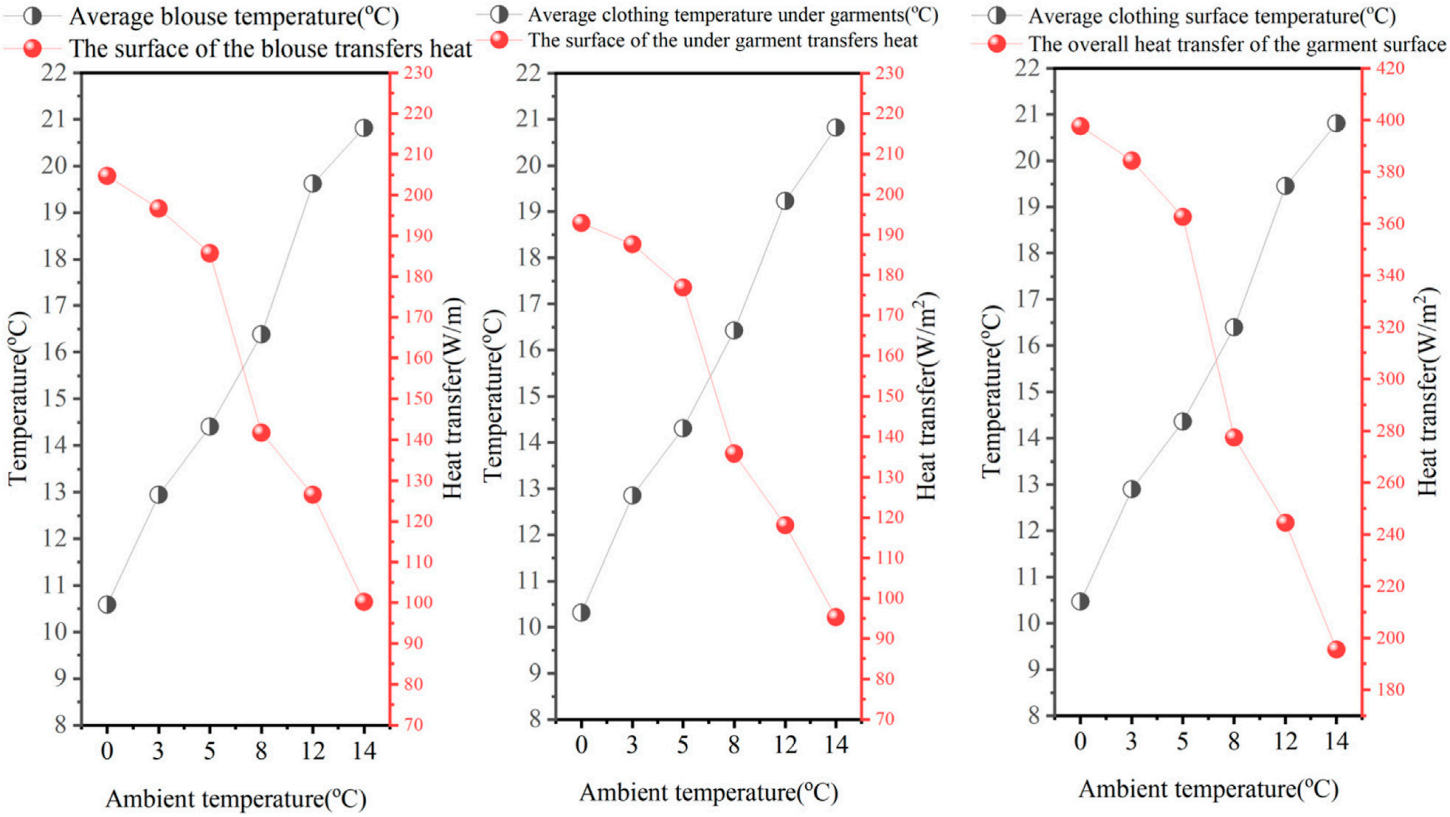
Simulated average temperature and simulated heat transfer on the upper body of the garment, the lower body of the garment, and the overall surface of the garment.

The average garment surface temperature values (upper body, lower body, and overall outfit) and garment surface heat transfer (upper body, lower body, and overall outfit) are shown in Figure 8. We can see that as the outside ambient temperature increases (top half of the garment, bottom half of the garment, and the garment as a whole), the temperature area-weighted average increases. When the ambient temperature increased from 0 °C to 5 °C, the overall garment surface temperature area mean increased by 2.35 °C. When the outside ambient temperature is low, the difference in the mean change in surface temperature of the garment increases significantly. The external ambient temperature values show a primary function toward the area-weighted average of the garment surface temperature. As the external ambient temperature increases, the surface temperature of the garment increases, and the difference between the human body’s core layer of 37 °C and ambient temperature decreases, and, ultimately, the body maintains a constant temperature. Clothing surface heat transfer varies less in relatively low-temperature environments and more in the 8 °C–14 °C range. This is because in low-temperature environments, the body will reduce the amount of heat dissipated to maintain a constant body temperature.

### 3.3 Skin surface temperature distribution and clothing surface temperature distribution

**FIGURE 9:**
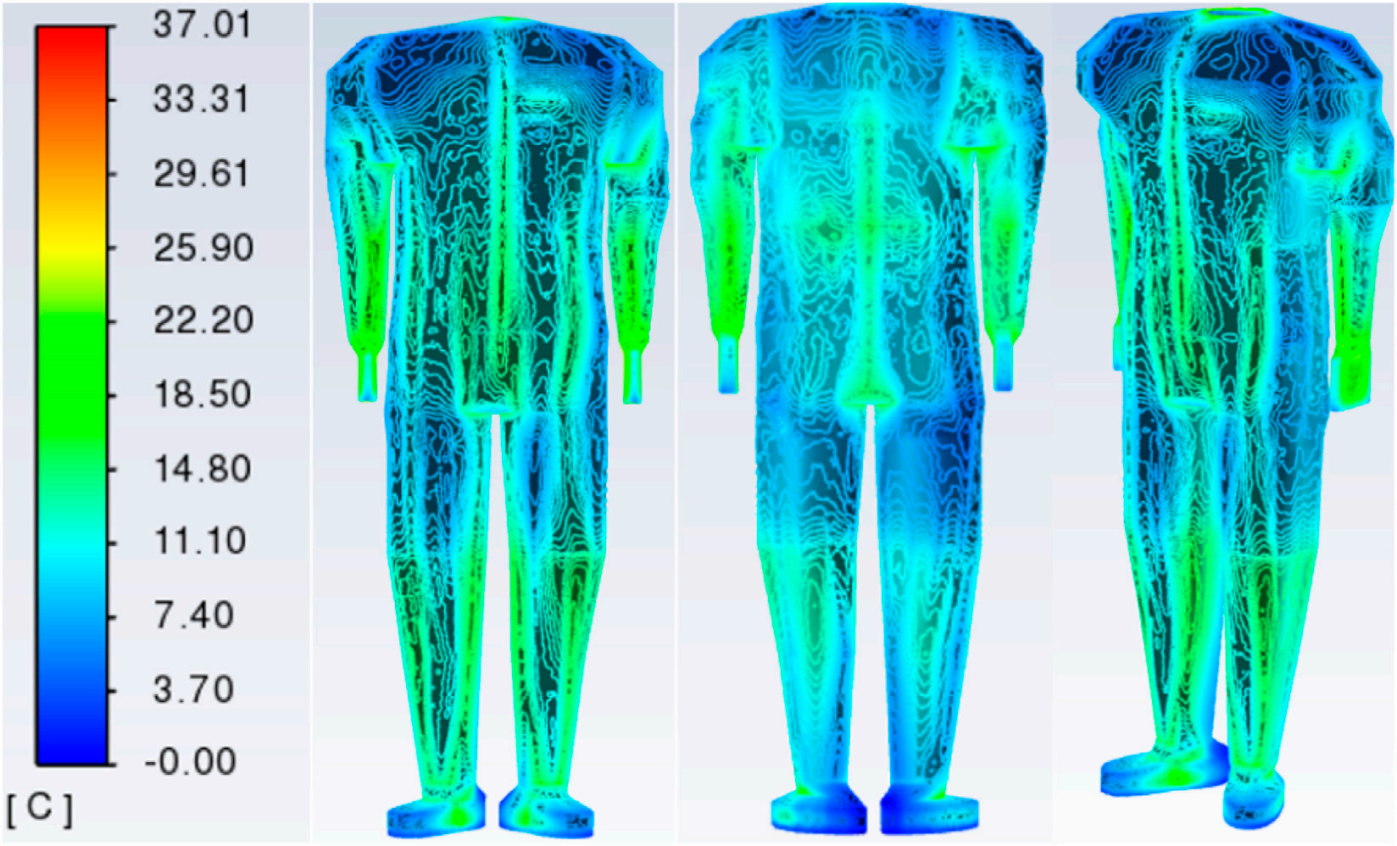
Cloud view of the temperature distribution of the front surface of the garment, the reverse surface of the garment, and the 45-degree viewing angle at an external environment of 0 °C.

**FIGURE 10:**
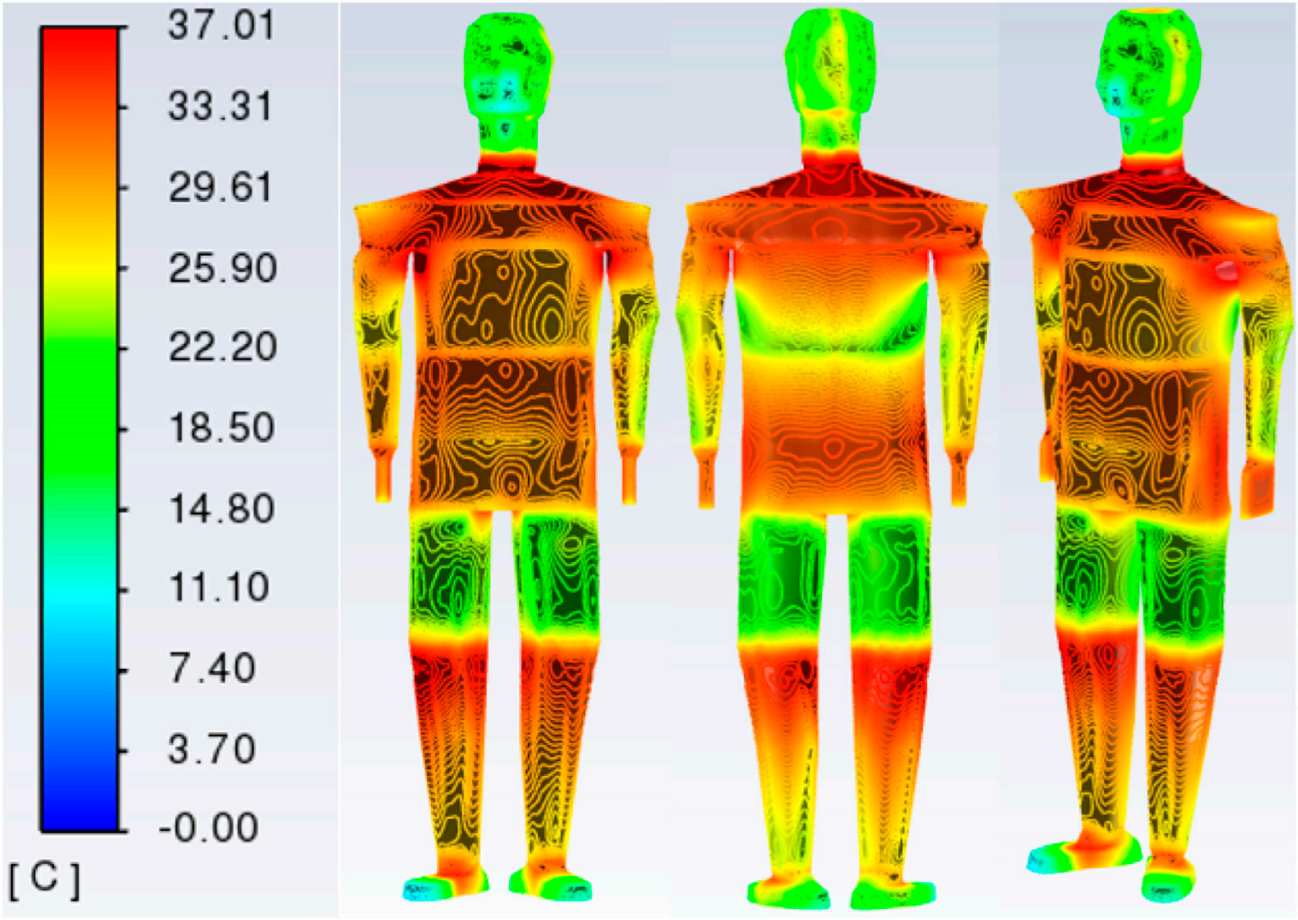
Cloud view of temperature distribution on the front side of the skin surface, the reverse side of the skin surface, and the 45-degree viewing angle at an external environment of 0 °C.

In Figure 9, we obtain the distribution of temperature values on the surface of the garment and the surface of the skin by simulation. The surface temperature distribution at an external ambient temperature of 0 °C is taken as an example. See Figures 9, 10. In Figure 9, the largest values of the garment surface temperature contour are in the upper body abdomen area. The lower arm and leg area will have a relatively large surface temperature of the garment due to the support of the bones, and the skin will fit tightly to the garment in these two areas. Due to the lower temperature of the external environment, the surface temperature values of the garments are smaller. At the ends of the limbs (toe tips, ankles, and hands), there is less blood flow, resulting in lower skin temperatures. In Figure 10, large values exist for ankle, hand, head, and crotch temperatures, which are covered by clothing in the human numerical model, resulting in relatively large thermal resistance and larger skin temperatures in these areas. In the crotch part of the body, skin is in close contact, so the skin temperature value in the crotch is larger. Skin temperature values are greater in the ankle area than in the toe area due to the greater thickness of the clothing layer. On the other hand, there is a stratification of the human skin surface temperature distribution, which is due to the fact that the 3D numerical model of the human body is divided one layer at a time. The size of the heat source of the equivalent layer in each part is different, so there is a difference in the distribution of the human skin temperature contour.

### 3.4 Equivalent layer heat transfer

The human body can be viewed as a thermal system that is capable of maintaining thermal equilibrium, that is, maintaining a stable skin temperature and core temperature, under certain external environmental conditions. Thermal homeostasis in the human body is the result of a series of complex interrelated physiological and physical interactions. This interaction includes vasodilation, vasoconstriction, sweating, shivering, and heat redistribution through blood convective heat exchange. Among other things, heat is exchanged between the skin and the external environment as a means of maintaining thermal balance. Humans are thermostatic organisms, and even with very large changes in the external environment, the body’s core temperature will remain near 37 °C, which is close to a constant state (Buse and Werner, 1985). In this article, the temperature of the core layer is set to 37 °C thermostatic wall surface to simulate the human body’s constant temperature characteristic.

The muscle and fat layers were stacked together to create an equivalent layer (Tanabe et al., 2002). Under ambient temperatures of 0–15 °C, calculated heat fluxes within the passive thermoregulatory system—including metabolic heat production, blood convection, external work, and tissue conduction—demonstrate significant magnitudes. These collectively serve as primary solid-phase heat sources within the equivalent layer. The derived heat source magnitudes are subsequently input into the equivalent layer framework. Concurrently, modulating the proportional contributions of individual heat components enhances the generalizability of the 3D human thermoregulatory model across diverse physiological scenarios. The calculated size of the heat source in the equivalent layer is entered into the equivalent layer at different external ambient temperatures. The real human experimental data were compared with the human numerical simulation results to finally obtain the range of equivalent layer heat source sizes for each part of the human body model at ambient temperatures ranging from 0 °C to 14 °C. The range of heat source sizes for the equivalent layers is shown in Table 5. For example, the head equivalent layer heat source size is $22.04W<Q_{head}<43.54W$ . In Tanabe et al. (2002), the head base heat flow was 17.17 W, the upper arm base heat flow was 0.639 W, and the abdomen base heat flow was 22.29 W. The difference between these areas and the base heat flow in Tanabe et al. (2002) was small. The volume average temperature values in the shoulder equivalent layer are in the range of 35 °C–36 °C, which is due to the core temperature value of the human axilla at 36 °C. It also shows that the 3D numerical model correlates well with the real human body model. It also verifies the reasonableness of the numerical model. In other parts of the human body in the equivalent layer, the volume average temperature values are also within 35 °C–37 °C, which is in line with the physiological characteristics of the human body.

**TABLE 5:** Range of equivalent layer heat source sizes for each part of the human body (W).

Body part	Head	Upper arm	Lower arm	Chest	Abdomen	R-thigh	L-thigh	R-calf	L-calf
Heat source size	22.04–3.54	0.63–2.35	2.27– 4.53	1.10–12.12	5.40–24.72	2.12–6.07	3.30–7.03	11.38–20.69	12.19–22.87

### 3.5 The effect of temperature differences in different external environments

The temperature difference was 3 °C, 5 °C, 8 °C, 12 °C, and 14 °C based on the human body at the external experimental ambient temperature of 0 °C. The experimental temperature measurements of the human head had the largest variation intervals of 4 °C, 7.07 °C, 5.5 °C, 7.88 °C, and 7.55 °C at different temperatures. This is due to the fact that the head is not covered by clothing, there is no insulation, and the head is more affected by the external ambient temperature and dissipates more heat. In the limbs, the temperature varied from 1.44 °C to 3.384 °C, and in the chest and abdomen, the temperature varied from 0.63 °C to 0.942 °C. It shows that the human limbs are more affected by the temperature difference of the external environment, and the shoulder, chest, and abdomen are less affected by the temperature difference of the external environment due to the fat wrapping and the higher blood flow in the area close to the heart.

## 4 Conclusion

This article establishes a 3D model of the human body based on scanned images of the real human body. The relationship between the human body, clothing, and environment is explored. The human body sweats less in a low-temperature environment. Therefore, skin evaporation heat dissipation is ignored, and the radiation and the gap between the clothing and the human body and other factors will be considered separately in subsequent work. In the skin temperature contour, the human body has higher values in the armpit and lower temperatures on the surface of the thigh skin, which is in accordance with the real human body model. The external ambient temperature values and the area-weighted average of the surface temperature of the garment basically show a primary function relationship. The inherent thermal resistance of the garment’s Ri, thermal resistance Rt, and the thermal resistance of the closed air layer Ra fluctuate less with increasing external ambient temperature, and Ri contributes more to the human body’s heat preservation. Finally, under the change of the external ambient temperature with a certain temperature difference, the limbs are affected by the ambient temperature, and the head is affected by the external environment the most due to the lack of clothing protection, with an average change value of 6.4 °C. The shoulders, chest, and abdomen are affected by the external ambient temperature less due to the proximity to the heart and the protection of the fat. The numerical simulation results show that the human body model has high reliability and accuracy. In the study of the human body’s heat dissipation mechanism, it also provides a numerical basis for other scientific research and engineering applications.

## 5 Limitations and expectations

The experiment described herein was conducted in a low-temperature environment, so the influence of human sweating was ignored. The insulation layer and evaporation layer of clothing are simplified to the clothing layer. The effect of wind speed will be added in future studies. Based on the low-temperature environment studied in this article, where the evaporative heat loss from sweat is minimal compared to metabolic heat production and other heat sources, the clothing model was simplified from a moisture resistance model to a pure thermal resistance model. Consequently, the effect of sweat evaporation was neglected. Future research could incorporate this factor to further enhance model accuracy, broaden its applicability, and address more extreme environmental conditions.

Building on the validated feasibility of the human numerical model, future work should include increasing the number of test subjects, extending the duration of skin temperature monitoring experiments, and exploring the long-term effects of low-temperature environments on human health and thermal adaptation. Additionally, research should investigate the dynamic interactions between the human body, clothing, and the environment under transient states.

## Data Availability

The original contributions presented in the study are included in the article/supplementary material; further inquiries can be directed to the corresponding authors.
